# Chancre of the Lip—Was It Communicated by a Dentist's Instruments?

**Published:** 1878-02

**Authors:** C. W. Dulles

**Affiliations:** Philadelphia.


### ARTICLE V.

### *Chancre of the Lip—Was it Communicated by a Dentist's Instruments?*

BY C. W. DULLES, M. D., OF PHILADELPHIA.

Some time since Dr. F. F. Maury gave me the opportunity of examining a patient under his care, with the following interesting and unusual history:—A. B., a female, aged twenty-six, is a thoroughly respectable housemaid, modest in her bearing, and her character vouched for by her employer, a physician, in whose family she has been for years. She says she has never had any serious illness, and her appearance is strongly corroborative of her statement. Although aware that she has now a grave disease, she does not know its specific nature.

In the month of September, when in perfect health, she suffered with a severe toothache, for the relief of which she applied to a dentist. He put some cotton, bearing an anodyne, into a cavity in one of her teeth, and advised her to have the nerve extracted and the tooth filled. A week later she returned, and he made an application to destroy the nerve. Still a week later he completed the operation, and recommended her to have all her teeth cleaned. To this she consented, and he did it.

At this time, two weeks from her first going to him, the angles of her mouth were much irritated by stretching, and she had observed a small sore near the middle of her lower lip. This the dentist said was only a blister, and advised the application of alum to it, which she carried out. However, the effect seemed evil, and the sore developed in about ten days into an angry, indurated ulcer, half an inch long, and of a somewhat less width, with a yellowish deposit, and an accompanying enlargement of a left submaxillary lymphatic gland. The ulcer and the bubo were both, she says, excessively painful.

Her condition now attracted the attention of the physician in whose family she was serving, who diagnosed a chancre, applied locally an ointment containing the nitrate of mercury, and gave her internally iodide of potassium and iodide of mercury, combined with opium.

The disease then passed on, with malaise and fever, to a general papular eruption, and the woman came, about five weeks after first going to the dentist alluded to, under Dr. Maury's care. She was, at the time of my first examination, ptyalized; on her lower lip was an enormous chancre, surmounted by an ugly excavated ulcer, an inch and a half long by about half an inch wide, with angry-looking, indurated and everted edges, and covered with a nasty yellowish deposit. Below the angle of the left lower jaw was a large and painful bubo. She had a general but not profuse papular syphiloderm. Many of the papules were developed in her palms and soles. On inquiry and examination there appeared to be no other syphilitic manifestations.

Under treatment the ulcer was, after two weeks, reduced one half; but there was appearing a very painful pustular syphiloderm. This began upon her shins and calves, and came afterward, though less severely, on her arms and face. Two weeks later she had a sore throat. The chancre was healing nicely. Later, this went on quite well, leaving but little loss of substance, and the bubo subsided almost entirely. The pustules, however were very troublesome. There were many of them, of various sizes and of different grades of ulceration, presenting the features of so-called syphilitic impetigo, ecthyma and rupia. Iritis seeming to threaten, instillations of atropia were used with success.

Without going further into the history, we have, then a case of syphilis, whose initial lesion was a chancre upon the lip. The important question is, how was the chancre contracted? The answer to this question involves so many delicate considerations that it may not be wise to express here a positive opinion in regard to it; but to entertain one, these points should be borne in mind: A woman of unimpeached reputation subjects herself to the manipulations about the mouth inseparable from a dentist's operations; about two weeks later there appears on her lip a lesion, the development of which is that of a typical chancre, and which is followed by other unmistakable manifestations of syphilis.

Bearing these facts in mind, whatever may be one's opinion in regard to the source of the syphilitic contagion and its manner of communication, the history of this case may well serve as a warning to all dentists, as well as to the medical profession. It will have special weight if considered in connection with the cases of inoculation from the virus of mucous patches in the mouth, recently investigated by Dr. Maury and the writer, and reported in the *American Journal of the Medical Sciences* for January, 1878, in an article entitled "Tattooing as a Means of Communicating Syphilis."

A prominent member of the dental profession in this city recently assured me he was constantly on his guard against the danger of conveying disease from one of his patients to another, keeping a special lookout for suspicious lesions. He has, he says, detected syphilitic lesions, and might, without this precaution, have inoculated them upon others. Such a course deserves to be followed by all; and perhaps it might not be amiss—if not already done—for the dental colleges to teach their pupils the diagnosis of syphilitic lesions in and about the oral cavity, impressing upon them the danger of inoculation and the means of avoiding it.—*Med. and Sur. Reporter.*

